# Ventilator-associated pneumonia due to *Aeromonas hydrophila:* A rare case report

**DOI:** 10.1099/acmi.0.000672.v3

**Published:** 2023-10-19

**Authors:** Amit Srivastava, Sarvodaya Tripathy, Shreyas Gutte, Chinmoy Sahu, Mohan Gurjar, Sangram Singh Patel

**Affiliations:** ^1^​ Department of Critical Care Medicine, Sanjay Gandhi Post Graduate Institute of Medical Sciences (SGPGIMS), Lucknow-226014, India; ^2^​ Department of Microbiology, Sanjay Gandhi Post Graduate Institute of Medical Sciences (SGPGIMS), Lucknow-226014, India

**Keywords:** *Aeromonas hydrophila*, critically ill, intensive care unit, ventilator-associated pneumonia

## Abstract

**Introduction.:**

*
Aeromonas hydrophila* is an opportunistic pathogen that can cause various infections, including pneumonia, in immunocompromised individuals. This case report presents a rare occurrence of ventilator-associated pneumonia (VAP) caused by *Aeromonas hydrophila* in an apparently non-immunocompromised patient.

**Case presentation.:**

The patient exhibited signs and symptoms of VAP and was successfully treated with intravenous ciprofloxacin. The discussion highlights the characteristics of *Aeromonas* species, its virulence factors, risk factors for infection, and antibiotic profile.

**Conclusion.:**

It emphasizes the need for awareness and suspicion of *Aeromonas* as a potential cause of VAP in ICU settings, as well as the importance of early detection and appropriate treatment for improved outcomes.

## Data Summary

All authors confirm that no data was generated or reused.

## Introduction

*Aeromonas hydrophila* is considered an opportunistic infection causing soft-tissue infections, gastroenteritis, and sepsis in immunocompromised hosts [1]. *Aeromonas* is an infrequently encountered microbial isolate in samples from the intensive care unit (ICU), often ignored as an incidental finding. It is multidrug-resistant and can lead to a fatal infection if left untreated [1]. Here we report a rare case of ventilator-associated pneumonia (VAP) due to *Aeromonas hydrophila *, in an apparently non-immunocompromised patient who responded to treatment.

## Case presentation

**Fig. 1.**
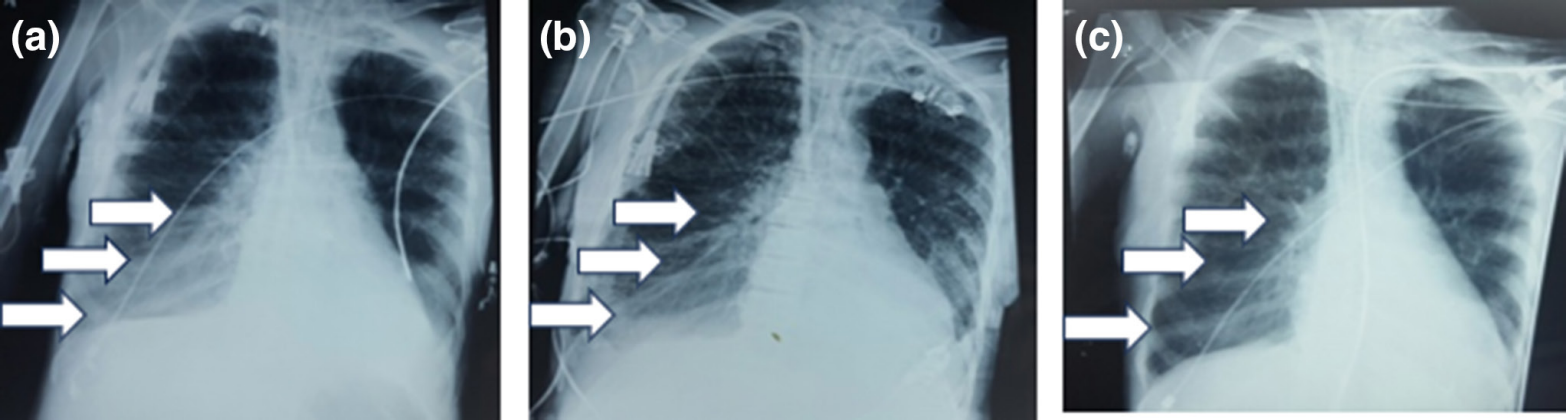
Chest X-ray (CXR) AP view of ventilator-associated pneumonia (VAP) (**a**) Arrow showing right lower zone dense patch of VAP on the eighty-first day of illness (DOI-81). Panel (**b**) and (**c**) showing resolution at (DOI-87) and (DOI-93) respectively.

A 65 year female, known case of hypertension and chronic kidney disease (CKD) on medical management, was admitted elsewhere [day of illness 1 (DOI-1)], with chief complaints of gradually progressive shortness of breath and decreased urine output. She was intubated (DOI-6) in view of the shortness of breath, and initiated on renal replacement therapy (RRT) owing to anuria and rising creatinine levels. She was shifted to our hospital (DOI-20) for further management. At admission to ICU, the patient continued to remain on organ-supportive therapies including invasive mechanical ventilation, vasopressor, and renal replacement therapy. During the clinical course, the patient remained on mechanical ventilation due to significant neuromuscular weakness and recurrent infections including VAP with different etiologies, *Pseudomonas aeruginosa* (at DOI-48), and *Klebsiella pneumoniae* (at DOI-74) which were treated with intravenous imipenem and meropenem with colistin, respectively as per antimicrobial sensitivity reports. On DOI-81, the patient again had sign and symptoms of new-onset infection with raised total leucocyte count (TLC 16000/cu.mm) and increased tracheal tube secretions, right lower lobe opacity in the Chest X-ray (CXR) (Fig. 1). High-resolution computed tomography (HRCT) thorax revealed diffuse centracinar and para septal emphysematous changes in both lungs likely infective (images not shown).

**Fig. 2.**
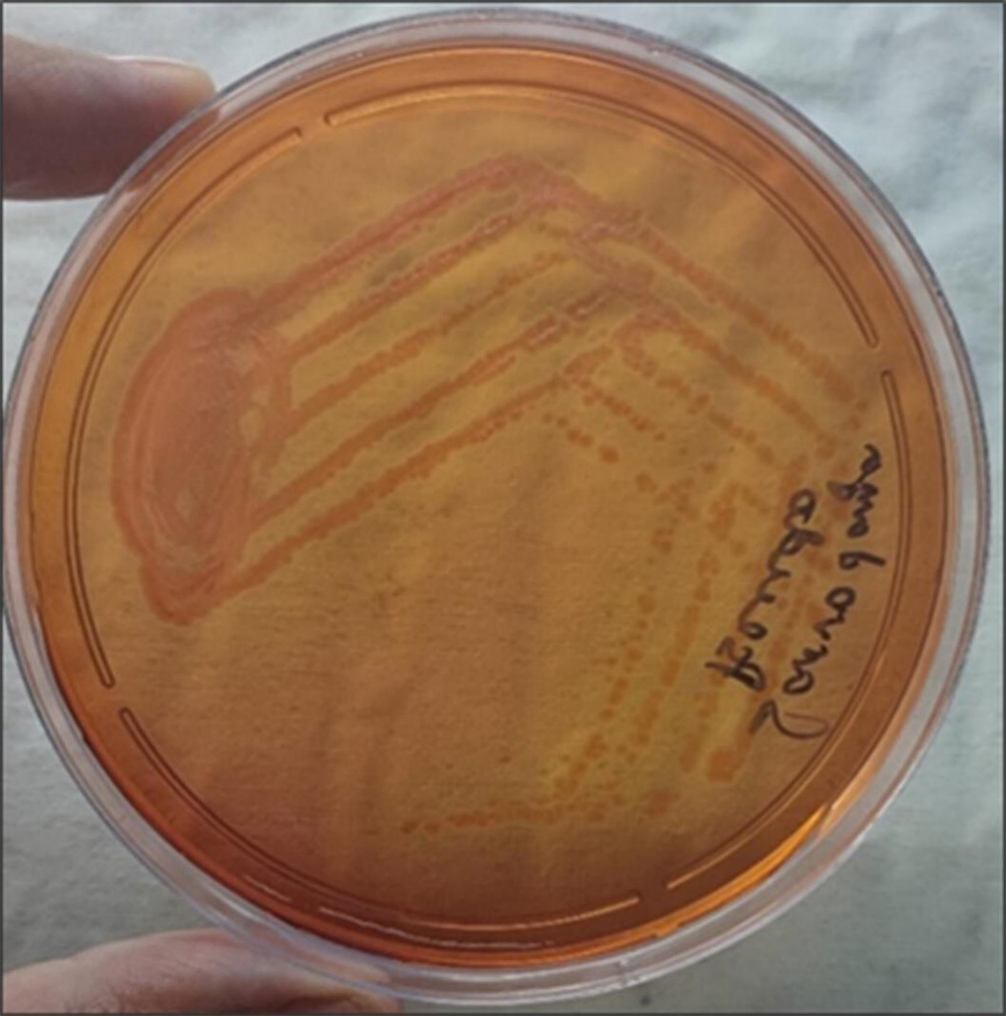
MacConkey Agar showing non-lactose fermenting colonies of *A.hydrophila*.

In microbiological processing of the sample obtained by bronchoalveolar lavage (BAL), direct staining from the BAL sample showed slender Gram-negative bacilli with pus cells. Culture was put up on Blood Agar and MacConkey Agar. On culture colonies were haemolytic greyish and circular on blood agar and showed non-lactose fermenting, flat, non-mucoid, spreading colonies on MacConkey agar that were catalase-positive and oxidase-positive (Fig. 2). It is a non-fastidious bacterium that grows well in ambient air after 16–20 h of incubation. Routine biochemical testing was done for presumptive identification of the isolate. Matrix-Assisted Laser Desorption Ionization Time-of-Flight Mass Spectrometry (MALDI-TOF MS) helped in pinpointing the species. The isolate was identified as *Aeromonas hydrophila *. The confidence level of MALDI-TOF for the detection of *Aeromonas* is 99 %.

Intravenous ciprofloxacin was started as per culture sensitivity, and the patient improved after 12 days of treatment (DOI-83 to 94), as evident clinically by a decrease in tracheal secretions, decreased TLC counts, and clearance of CXR opacities (Fig. 1). Subsequently, the patient was weaned off from the ventilator and discharged to home (DOI-120) in a clinically stable condition with continuing domiciliary care as per family members request with advice for continuing maintenance hemodialysis (twice weekly frequency), tracheostomy tube (due to inadequate power to clear respiratory secretions) and other supportive nursing care. In telephonic follow-up, family members informed about the death of the patient after 4 weeks of ICU discharge, however, family members did not reveal details about the terminal events.

## Discussion

*Aeromonas* are facultatively anaerobic, Gram-negative bacilli, which are oxidase- and catalase-positive [1]. Initially, this genus was included in the family *Vibrionaceae* but at present put in a separate family *Aeromonadacae* based on deoxyribonucleic acid (DNA) hybridization and sequence analysis of 16S ribosomal ribonucleic acid (rRNA) studies [2]. *Aeromonas* species are frequently isolated from fresh or brackish water, sewage, soil, and tap water in temperate or subtropical countries [3]. Chlorinated water can also be containing *Aeromonas* [4].

*Aeromonas* has been regarded as an opportunistic agent that mainly causes gastroenteritis. Extra-intestinal infections caused by *Aeromonas* spp. include soft-tissue infections, arthritis, endocarditis, meningitis, urinary tract infections, pneumonia, and even sepsis in immunologically compromised hosts [5, 6]. The majority of *Aeromonas *-associated pneumonia has been reported after freshwater or saltwater near drowning [7–9]. Though rare, it has been reported in empyema, lung abscesses, community-acquired as well as nosocomial pneumonia in immunocompromised patients [10]. Malignancy and diabetes mellitus have been found to be common risk factors [5, 11]. Other significant risk factors include alcohol abuse and related hepatic disease, cardiovascular, cerebrovascular diseases, and chronic lung infections [12]. In our case, prolonged illness with the need for organ supports like mechanical ventilation, renal replacement therapy, antimicrobial exposure were risk factors present for development of *Aeromonas* associated VAP.

Its involvement in nosocomial pneumonia has garnered attention due to its ability to colonize and persist in the respiratory tract. Cross-contamination due to improper hand hygiene, contaminated water used for humidification of airway tubings, and direct hematogenous transmission from the gut to lungs are major sources of *Aeromonas* pneumonia in critically ill patients. Additionally, for developing pneumonia, prolonged mechanical ventilation, underlying lung diseases, prior antibiotic therapy, and exposure to contaminated water sources are important risk factors in ICU settings. However in our case, ecological study was not carried out to rule out source. Also, no other cases had *Aeromonas* VAP during same time period in the ICU.

Virulence factors present in *Aeromonas* include lateral flagella, pilli, extracellular proteins like proteases, lipases, exotoxins, etc. [13]. These factors aid in its colonization and invasion of the respiratory tract. Furthermore, its ability to form biofilms on endotracheal tubes and other medical devices contributes to its persistence in ICU patients. Studies have revealed infection with *Aeromonas* cause hypoxia-induced organ damage to severe necrotizing pneumonia with signs of pulmonary oedema, on histological examination. This is suggestive of the virulence of this pathogen and could explain the rapid clinical course and high mortality rate reaching up to 50 % as reported in the literature [14, 15].

*Aeromonas hydrophila* VAP presents with symptoms similar to other types of VAPs, including fever, cough, purulent sputum, and radiographic evidence of pneumonia. Diagnosis typically involves obtaining respiratory samples for culture and identification of the organism. In our case, we could isolate the organism from the BAL sample which is the best possible minimally invasive respiratory sample and a very reliable one.

*Aeromonas* spp., including members of *A. caviae, A. hydrophila*, and *A. veronii* complexes, constitute the majority of susceptibility testing data. European Committee on Antimicrobial Susceptibility Testing (EUCAST) recommends cefepime, ceftazidime, aztreonam, ciprofloxacin, levofloxacin and cotrimoxazole [16]. Clinical and Laboratory Standards Institution (CLSI) recommends testing with piperacillin-tazobactam, cephems, carbapenems, aztreonam, aminoglycosides, cotrimoxazole, and fluoroquinolones for *Aeromonas* [17]. *Aeromonas* strains are inherently resistant to ampicillin, amoxicillin-clavulanate, and cefazolin [18–20]. Some strains may develop resistance due to inducible β-lactamases during treatment. Resistance to carbapenems can occur, colistin resistance has been reported due to *mcr 3* gene [21]. The Infectious Diseases Society of America (IDSA) recommends a combination of doxycycline with ciprofloxacin/ ceftriaxone for extraintestinal infections [22]. Ciprofloxacin breakpoints include ≤0.25 mg l^−1^ as sensitive and >0.5 mg l^−1^ as resistant as per EUCAST [16]. Treatment should be guided by clinical response [22], which was done in our case. Reasonable courses of therapy include 2 weeks of therapy for treatment of systemic infection. We used Kirby Bauer’s disc diffusion method of antibiotic susceptibility testing. The isolated strain in our case was susceptible to amikacin, ciprofloxacin, ceftriaxone, ceftazidime, cefoperazone-sulbactam, imipenem, and meropenem. It was resistant to colistin. Ciprofloxacin is a good agent for pneumonia and the isolate we obtained was sensitive to it. So, the patient was treated with intravenous ciprofloxacin.

It has been estimated that the worldwide incidence of *Aeromonas* infection in humans varies in geographical location and developed to underdeveloped nations, ranging from 0.66 cases per million to 15 cases per million [14, 23, 24]. Indian studies identified *Aeromonas hydrophila* in more than 30 % of the fish population under study [25]. There are case reports of different species of *Aeromonas* causing VAP [26–29], single case of *A. salmonicida* has been reported in a study, and even a case of community acquired aspiration pneumonia due to *A. hydrophila *. Cases of *A. hydrophila* pneumonia due to leech therapy have also been documented [30].

## Conclusion

Although it has long been known as an aquatic pathogen, *Aeromonas hydrophila* has become a pathognomonic contributor to VAP during prolonged illness. It is necessary to be aware of the possibility that *A. hydrophila* as the cause of VAP in the ICU settings. For managing *A. hydrophila *-associated VAP, a high index of suspicion, and focused antimicrobial stewardship with avoidance of contaminated sources are important, given the particular resistance pattern of the bacterium and high mortality rate. Several variables, like the patient’s underlying health status, the promptness of the diagnosis, and the suitability of the antimicrobial therapy, can affect the prognosis of *A. hydrophila* VAP. If the infection is not properly treated, complications like sepsis, respiratory failure, and multi-organ dysfunction syndrome may develop. However, positive outcomes are possible with early detection, adequate treatment, and supportive care.
